# Chemistry and Production Technology of Hallstatt Period Glass Beads from Bohemia

**DOI:** 10.3390/ma15165740

**Published:** 2022-08-19

**Authors:** Zuzana Zlámalová Cílová, Viktoria Čisťakova, Romana Kozáková, Ladislav Lapčák

**Affiliations:** 1 Department of Glass and Ceramics, Faculty of Chemical Technology, University of Chemistry and Technology Prague, Technická 5, 166 28 Prague, Czech Republic; 2 National Museum, Václavské náměstí 1700/68, 110 00 Prague, Czech Republic

**Keywords:** glass beads, Hallstatt period, Bohemia, Raman spectroscopy, SEM/EDS, LMG, antimonate opacifiers

## Abstract

The presented study evaluated a set of beads primarily originating from the Hallstatt period (800–400 BC) and uncovered in the region of Bohemia. Utilizing an SEM/EDS method, the chemical composition of the glass samples was determined and their homogeneity measured. Owing to the presence of opaque glass, Raman spectroscopy was applied, enabling the definition of the phases causing the opacity of the glass, as well as its coloring. This article discusses opacifying agents, including the possible ways in which they entered the artefacts. In addition, the techniques used to produce the glass beads are described, for both the single-colored beads, as well as the so-called eye beads that are present in a significant amount in the set. The majority of the beads examined were found to be made of the LMG glass type (low-magnesium soda-lime glass). An unexpected result was the identification of glass with a high content of K_2_O not corresponding to the mixed alkali type (LMHK), which is frequently discussed in the literature. The glass type in question most likely does not come from the traditional area of glass production: the eastern Mediterranean territory.

## 1. Introduction

**Figure 1:**
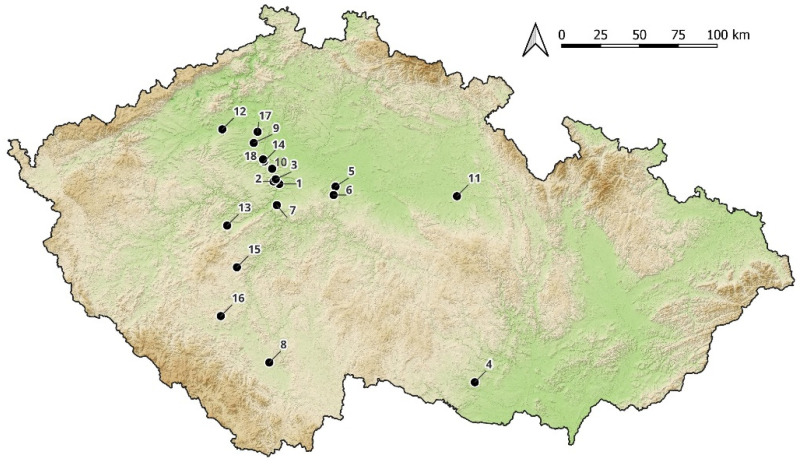
Map: 1. Prague, Nové město, 2. Prague, Střešovice, 3. Prague, Bubeneč, 4. Křepice hillfort, 5. Bylany, 6. Chotýš, 7. Lhota-Závist hillfort, 8. Mydlovary, 9. Černuc, 10. Roztoky-Žalov, 11. Platěnice, 12. Pátek, 13. Lochovice, 14. Holubice, 15. Rtišovice, 16. Láz, 17. Straškov-Vodochody, 18. Minice hillfort.

**Figure 2:**
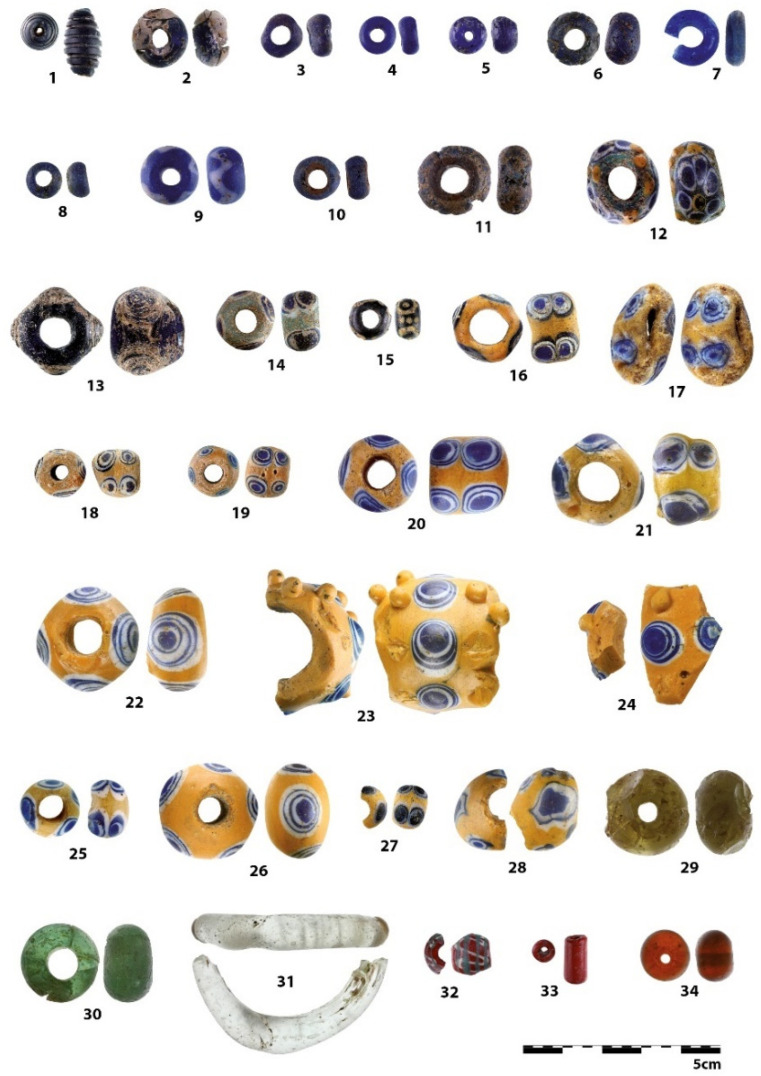
Analyzed glass beads with sample numbers (see Table 1 for Inv. No. and site information). National Museum in Prague collection, Archaeological Institute in Prague collection, UAPPSČ collection, Roztoky Museum collection. Photo Martina Košařová.

The archaeological collection of the National Museum in Prague undoubtedly has a rich history. Its first collections were formed as early as the 18th century, often coming from the private collections of the nobility or amateur excavations. However, they sometimes lack basic documentation [1]. One of the main reasons that led to an investigation into the elemental composition of the glass beads coming from the collections of the Hallstatt period was the need to specify the chronological dating and identify the more recent finds. From this standpoint, the most problematic item to analyze was a set of four beads allegedly coming from the prehistoric hillfort of Křepice (beads numbered 1, 5–6, 34; Figure 1 and Figure 2 and Table 1), where archaeological research was carried out at the end of the 19th century. Considering that a number of prehistoric cultures are represented at the site, and in addition to the issues with the original research methodology, it can be assumed that older or younger intrusions occur among the Hallstatt period objects [2].

The beads from other sites (mostly from grave contexts) were primarily selected on the basis of typological variability, while their production specifics or manufacturing defects were also investigated. To supplement the existing collection, beads from several recently examined settlement sites, namely Chotýš and Holubice, were used. In the second stage of the research, a set of nine beads from the Late Hallstatt/Early La Tène hill fort of Lhota-Závist was added to the study.

Another objective of the research was to complement the general data on the elemental composition of glass beads and to investigate the specifics of their production in the Hallstatt period, as the chemical and technological research of the glass beads from the Hallstatt period has long been marginal in the Czech Republic. We can mention an essential piece of work devoted to this issue by Frána et al. [3], which focused on the analysis of the elemental composition of prehistoric glass, including beads from the Hallstatt period. Subsequently, as far as Czech prehistoric glass objects are regarded, N. Venclová, in her publication “Prehistoric glass” in Bohemia [4], created a fundamental system for the typological determination of individual artefacts, and this system was employed in this article (data in Supplementary form including references [5,6,7,8,9,10,11,12,13,14,15,16,17,18,19,20,21,22,23,24,25,26,27,28,29]).

## 2. Prehistoric Glass

The earliest glass of the Bronze Age can generally be classified into two groups, namely high-magnesium glass (HMG with 8–20% Na_2_O, 0–3% K_2_O, 2–10% MgO and 3–10% CaO; 1500–800 BC) and the later occurring low-magnesium and high-potassium glass (LMHK with 0–8% Na_2_O, 4–18% K_2_O, 0–1% MgO and 0–4% CaO; Final Bronze Age) (see reference [30]). The so-called primary production workshops of HMG are believed to have been located in Egypt, Mesopotamia, Anatolia, Greece, and south-western Iran. From these workshops, glass ingots were imported into the Mediterranean region, where they were further processed into their final forms in secondary workshops. Artefacts made of HMG have been uncovered in several regions in Europe, e.g., in Italy, Poland, Germany, and France [30,31,32].

In the European context, the production of glass beads dating to the Final Bronze Age (1200–1000 BC) is rather unique and is associated with the north-eastern part of Italy and the Frattesina region. Glass produced in this area is classified as LMHK and belongs to the so-called mixed alkali glasses. Glass of this type appears to be quite widespread in Europe, with finds documented from Switzerland, Germany, France, England, and Greece [33,34,35], but also from the region of Bohemia [5]. More recent results of glass analyses [36] indicated that LMHK glass was not produced by just one workshop, but other Italian workshops are also suggested as a source (note: here, it is worth mentioning that LMHK glass has no recognized chemical counterpart in the Middle East).

Both of the glass types described (HMG and LMHK) are assumed to contain the addition of plant ash applied in the form of an alkaline raw material. However, LMHK glass is richer in its content of K_2_O, and the introduction of alkaline leached wood ash has also been suggested [35]. The literature [30] also mentions the occurrence of these types in relation to different regions of Europe. For example, HMG glass seems to have been present in central and northern Europe for a longer time than in Italy. Both of the types, HMG and LMHK glasses, have also coexisted here for a longer period of time than they have in southern Europe.

From a chronological view, another chemical type is low-magnesium glass (LMG with 13–20% Na_2_O, 0–1% K_2_O, 0–1% MgO and 5–10% CaO; after 800 BC). The work of Purowski et al. [37,38] states that the LMG type started to replace HMG as early as the 9th or 8th century BC, while Conte [36] even mentions the 10th century BC. The LMG has low K_2_O and MgO contents, mostly reaching below 1.5%, and the alkaline raw material applied is natron (mineral soda). Concerning the later period (Late Hellenistic–early Roman period), the production of natron glass is assumed to have been mainly concentrated in the so-called primary centers, from where the raw glass in the form of ingots was transported to other workshops [36]. A production model for the period from ca. 10th century to the 1st century BC has not yet been proposed (as relevant production centers are not documented archaeologically).

However, glassmaking is known to have developed in Syrian Palestine and Mesopotamia in the earlier Iron Age (8th–6th centuries BC), when both small glass objects and glass vessels formed on a clay core or mold were produced. Other workshops were probably established in the Adriatic region, in the territory of present-day Slovenia, and on the island of Rhodes. Glassworks producing small objects also existed in the area of the northern Black Sea. The finished products then spread to Central and Western Europe as well. An interesting example is a set of glass finds and even faience beads from the cave Býčí skála, found in Moravia. The total number of beads uncovered is estimated to have exceeded 4500 pieces, which makes the set the largest collection of glass objects from the Hallstatt period north of the Alps. It is possible to infer the existence of a separate workshop in Central or South-Eastern Europe, but there is no evidence for this. In the 6th and 5th centuries BC, the Syrian–Palestinian coast remained one of the main centers of glass production. Here, in the Phoenician workshops, both glass vessels and small decorations such as beads were made.

In certain areas (Mesopotamia, Iran, and Central Asia), the production of HMG glass continued (here, a complete change in raw materials had not yet happened, i.e., the replacement of plant ash with natron). The HMG type is also recorded in several European regions, e.g., in Poland [37] with finds from the early and full Iron Age, in the HaC–early HaD (~the 8th to the end of the 6th century BC). This confirms the above-mentioned information about the coexistence of several types of glass in the given period.

The aim of the study presented here was to determine the chemical composition of glass beads from ca. 700–450 BC and to distinguish their possible subgroups. Owing to the coloring of the beads analyzed, individual colorants and glass opacifiers are also discussed. Finally, the data obtained are compared with the available literature.

## 3. Methods

An initial survey was carried out using optical microscopy before, during, and after the conservation of the individual beads. The beads were observed under different types of illumination using a Keyence VHX-S600 microscope (Mechelen, Belgium) with a high level of resolution, which enables one to view the rugged surface at higher magnifications. Lateral illumination highlighted the surface changes that had occurred due to the corrosion damage of the inhomogeneous glass. These changes often correspond to the production technique or the direction in which the molten glass was shaped. By observing the corroded surface, it was possible to identify non-preserved decoration and to determine how it was applied to the surface. Damaged beads were also examined on their fracture edges, where the illumination of transmitted light was primarily applied, enabling one to observe unmelted parts, bubbles, and other inhomogeneities inside the glass mass.

To analyze the original glass, the surface of the beads examined was polished (with diamond sprays to a final diameter of 0.25 µm) to remove corrosion products and other impurities. Owing to the non-conductivity of glass materials, the polished surface was covered with a carbon layer for the subsequent analysis. The samples were examined using a Tescan Vega3XMU scanning electron microscope equipped with a microanalytical system with a silicon drift detector (SDD) from the Bruker company and a Quantax 200 energy dispersive analysis system. The operating conditions for the analytical work were as follows: accelerating voltage 15 kV, working distance 15 mm, total analysis time of one measurement 120 s. To ensure the reliability of the measurements, the analysis was performed on several selected platforms. Opacifiers or other inhomogeneities were analyzed using a point analysis. The spectra measured were quantified using a ZAF-type correction procedure, and the images acquired were scanned with a backscattered electron (BSE) detector. The accuracy and precision were monitored with secondary standard reference materials (Corning Glass A, Corning Glass B) with an accuracy of 5% or better for each chemical element.

Raman spectra were measured with a Raman dispersive spectrometer-model DXR Microscope from the company Thermo Scientific (Waltham, MA, USA). The device was equipped with an Olympus confocal microscope. The excitation source used was a diode-excited Nd:YAG laser with a wavelength of 532 nm and an input power of 10 mW. A grid of 900 lines/mm was applied. A multichannel thermoelectrically cooled CCD camera was utilized as a detector. Samples were measured at 50× magnification with a measurement trace of approx. 1 µm^2^ and through the aperture of a 50 µm pinhole. Measurements were performed with a power of 3–10 mW (depending on the sample stability), a measurement time of 5 s, and 10 spectra accumulations. The study of the crystalline phases was further performed with a micro *X*-ray diffraction analysis of the sections that had originally been polished for SEM/EDS analysis. A D8 Discover micro-diffraction system was used with a Co lamp, and radiation at 45 kV and 30 mA. Angles from 10 to 119° 2θ were scanned, with a step size of 0.01° 2θ and 300 s scan step time. Finally, the data were evaluated using the PDF-4+ database.

## 4. Results and Discussion

### 4.1. Optical Properties, Production Technology

The beads examined were made of a wide variety of glass, including translucent and opaque materials with blue, greenish, blue-black, terracotta red, green, white, and yellow colors. However, they were all produced using a common shaping technique, namely winding. The only exception was the part of the ring made of clear glass that was shaped manually. In general, winding is one of the oldest and longest-used techniques to produce glass objects.

**Figure 3:**
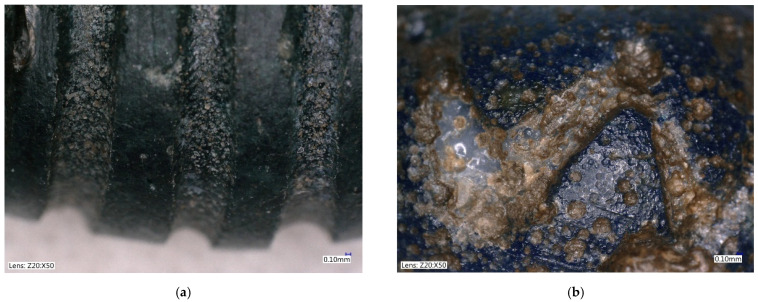
Beads with unpreserved decoration. (**a**) Bead No. 1, residual traces with bubble structure; (**b**) Bead No. 6, remains of light blue fiber.

An issue addressed in several studies is the method used to pick up molten glass. According to A. Hodgkinson [39], pre-prepared glass rods were already used in ancient Bronze Age Egypt (ref. [39]—Figure 3, ref. [40]—Figure 1). The glass rod was heated up with a fire torch in order to form a gather: a hot ball of glass at the end of the rod. The gather was then attached to a metal rod, which was rotated horizontally in order to produce a bead. Another way of shaping glass is to collect a glass fiber directly from the melt placed in a small crucible [41] using a metal rod, or by putting it directly onto a support rod.

The size of the shaft, being determined by the thickness of the metal rod used, is highly variable in the set analyzed. It ranges from 1 to 12 mm, with the greatest size variations seen in the eye beads. The shaft is equally large or slightly tapered at both ends of the bead. The nature of the shafts is in agreement with those of wound representatives from a Roman glass study [42]. In the wound beads examined, residues of clay materials or a granular structure were always present in the shafts. It can be assumed that various types of clay were already used as separators (melt/carrier rod) at this time. The specific type of separator cannot be reliably identified in the archaeological finds contaminated with soil, but the use of lime can be ruled out, as suggested by the study [42], as its addition would result in an increased content of calcium.

The blue beads from the set (Nos. 3, 4, 7, 8) were produced by simply winding a thicker thread. In beads 2 and 5, multiple-centered winding of a thinner thread is visible, just as it is in beads 29–30. Significant reaming of the wound layers is evident in the translucent part or in the case of major corrosion damage. As regards the amber brown bead, No. 34, the use of a mold to obtain a spherical shape can be considered, due to the way the surface is scratched.

Rounded bead No. 6 and fusiform/cylindrical bead No. 1 both belong to the category of beads with colored decorations, although their decorative lines were not preserved physically. At first glance, both beads seem to have been decorated with an engraved line, but in fact they were decorated with a fused fiber of a different color. This may have selectively corroded, and possibly cracked and peeled away, leaving only residual traces with a bubble structure, due to hot bonding (Figure 3a). Similar bubbles at the interface of fused glass can be observed, for example, in the eye beads Bead 6, on closer observation, displays the remains of a light blue fiber, whose condition was strongly affected/damaged by corrosion processes (Figure 3b).

**Figure 4:**
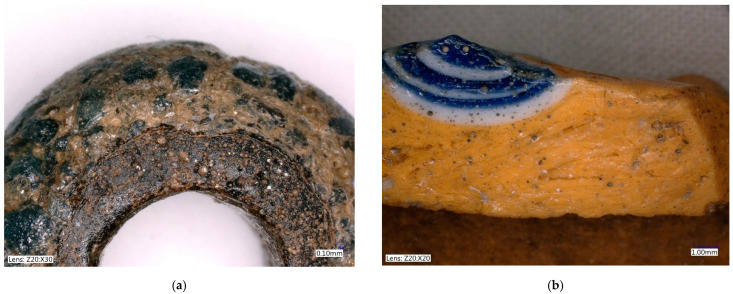
Inhomogeneity of examined beads. (**a**) Bead No. 11, double layer structure; (**b**) Bead No. 23, apparent direction of shaping.

In the context of the examined collection, the two-layer beads Nos. 10 and 11 are rather atypical specimens. The core of bead No. 11 is formed by dark blue to black glass with distinct bubbles and a number of macroscopically visible unmelted particles. On its top, a thick layer of a lighter blue color is deposited, again with visible inhomogeneities, but in much lower amounts (Figure 4a). The upper glass is badly damaged by corrosion, which has caused deep cracks in the entire layer, locally accompanied by other small hair-like cracks. The degree of damage could also have been influenced by the different dilatation of the chemically distinctly different glasses used, which caused the cracking of the outer glass, and thus the resulting cracks could have been more significantly affected by corrosion mechanisms. In contrast, bead No. 10 is almost intact. A possible reason for this lies not only in its more uniform composition, but also in it having a notably thinner inner glass layer. The frequent cracking of multi-layered glass is likely the reason why most archaeological contexts lack it. Similarly produced beads have been more frequently observed in Late La Tène sites, when the technology may already have been more advanced [43].

The production technology of the largest group, that of the yellow beads with eyes (Nos. 16–28), proved to be uniform. By layering white and blue glass, a colored eye was created. Owing to the inhomogeneity of imperfectly mixed glass, the direction of shaping can be observed (Figure 4b). The internal structure of basic yellow glass corresponds to the rolling of a larger amount of glass from the melt directly around a metal rod, or pouring it into a mold with an inserted rod [44] rather than to the multiple winding of a thin glass filament or the use of a pre-prepared rod. The eyes, on the other hand, were probably produced by applying a pre-fused glass rod, as the layers of matching colors always have an identical composition. In the case of scooping melted glass from a crucible, a completely identical structure and composition cannot be achieved readily, due to the inhomogeneity of the melt. The hot glass creating the individual eyes had to be pressed into the surface quite strongly with either a metal tool or by applying pressure on a pad. This can be seen, for example, in the cut of the massive bead No. 21, where reams of yellow glass occur around the fused eyes. The plastic eyes on this bead were applied last as hot prunts, not by squeezing with pliers or scissors on the base bead.

### 4.2. Chemical Composition of the Glass

**Figure 5:**
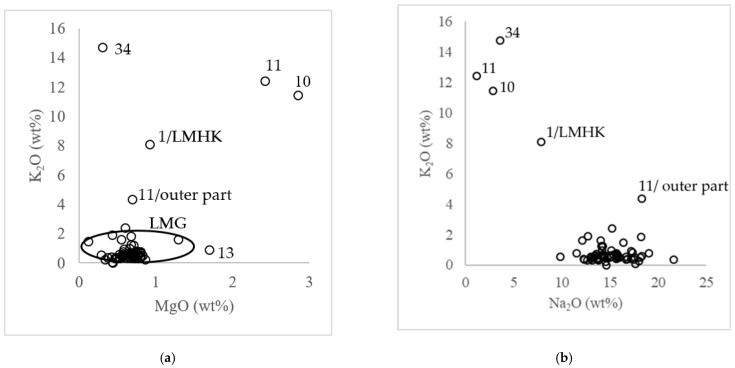
Plots: (**a**) K_2_O compared to MgO in the analyzed samples; (**b**) K_2_O compared to Na_2_O in analyzed samples.

The criterion used for the basic division of soda glasses is the content of MgO and K_2_O. Glasses are referred to as natron if the contents of the mentioned oxides are up to approx. 1 wt.% (Table S1); however, there exist wider limits for these values [38,45,46]. The samples analyzed in our work are primarily of the sodium natron or LMG type (see Table S1 and Figure 5a). This claim can be supported by the confirmed presence of SO_3_, with the measured amounts of Cl (0.3–1.3%) and P_2_O_5_ being mostly below the detection limit. Concerning the LMG glass, the following values can be reported for the most prevalent oxides: SiO_2_ in the range of 57–72% (with a predominant value of about 65.5–68%) and Na_2_O with most values in the range of 13–19% (Figure 5b).

The LMG group can be expanded to include the LMMK group (low-magnesium and medium-potassium). Several works [37,38] define this group as containing a maximum amount of MgO of 1.5%, but having a higher amount of K_2_O. A range of about 1.4–2.2% for K_2_O is reported by Purowski [37], which was confirmed in our data set, e.g., for blue bead No. 3 with a K_2_O value of 1.84%.

The LMMK glass is usually considered to be of inferior quality, with a number of imperfections and bubbles. Therefore, it seems rather unlikely that its production came from the Middle East and Egypt, with the territory of Europe having been suggested as an alternative. The LMMK glass included the glass samples dated to Hallstatt C (c. 750/700 to 600 BC), while Hallstatt D glass (c. 600 to 400 BC) was classified as LMG [37].

The samples that significantly disagree with the criterion for LMG of K_2_O and MgO contents being up to 1.5% are 34 (transparent orange bead), 1 (fusiform/cylindrical bead in blue), and 10 and 11 (inner layers of double-layered blue beads). Sample 13 only displayed a higher MgO content (bead with single-colored spiral decoration), while only a higher K_2_O content was detected in the blue segment of bead 27 and 3, and in the outer glass of double-layered beads 10 and 11.

A significantly increased amount of K_2_O was found in bead 34 (14.7%; Figure 5a and Table S1) accompanied by a relatively low MgO content (0.3%), but, in this particular instance, phosphorus was not detected. On the basis of these data, it is possible to exclude the use of raw ash as an alkaline raw material and infer the application of a potassium raw material of a potash type instead. The production of potash, for example, is documented in Bohemia as early as the Middle Ages, but it was not until the Baroque period that it became more widely used in glassmaking. A small amount of Na_2_O may have been introduced by salt (NaCl), which can also be seen in Bohemian early modern glass [47]. Although the bead under examination was not dated unambiguously, judging by its overall composition, it is likely that it is an artefact more recent than the other beads in the collection.

Blue bead 1 can be assigned to the group of LMHK type glasses. As already mentioned above, the production of these glasses is associated with the Fratessina site, and the criteria defining this type of glass can be found in the literature [33]. The following factors are typical of these glasses: (a) specific alkaline composition, with an almost constant total amount of K_2_O and Na_2_O (13–16 wt.%), (b) MgO content always below 1 wt.% and proportional to the CaO content at a ratio of 1:2, and (c) FeO content linearly proportional to that of Al_2_O_3_ at a ratio of 1:3. On comparing these factors and the results of the analysis of the bead examined, it is clear that sample No. 1 belongs to this group. In addition, particles with a high SiO_2_ content were found in the bead mass, and a relatively high CuO content was detected as well, which corresponds with another description of this glass type as found in the literature [33]. The type of LMHK (*mixed alkali*) glass was previously described in connection with our territory and the Late Bronze Age in [5], for instance.

Other somewhat atypical samples are beads 10 and 11, both of a similar structure. The beads are made of two types of glass, where the inner, highly heterogeneous, glass is overlaid with blue glass, which is almost homogeneous. A structurally similar type is described by Purowski [38] in the form of beads made of glassy faience decorated with true glass from the Hallstatt C period (c. 750/700–600 BC) in Poland. Glasses utilized for the so-called glassy faience are defined in [36] as two types: LMMK (K_2_O average at 2.7%) and LMGGF (natron-based, with a lower K_2_O content but higher levels of PbO and Sb_2_O_5_). The glass referred to as “true glass” is of the LMG (i.e., natron) type, again with higher contents of PbO and Sb_2_O_5_, which is related to its opacity (yellow glass).

**Figure 6:**
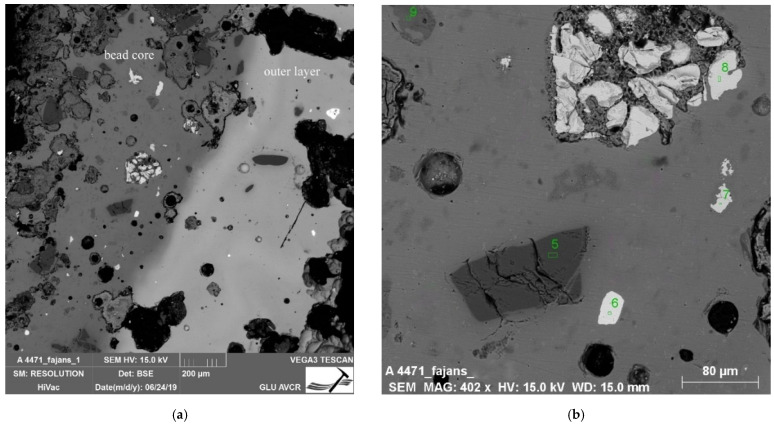
(**a**) Layer stratigraphy of Bead No. 10; the darker heterogeneous structure (to the left) represents the bead core with a high K_2_O content, while the shell of the bead with a more homogeneous structure can be seen on the right; (**b**) Detail of image (**a**) showing the bead core/internal layer. The glass mass contains SiO_2_ (5) and Zr particles (6), iron-rich phases (7 and 8), and most likely feldspar grain (9).

The significant difference in the glass mass forming these samples (10 and 11), besides the quality/homogeneity (Figure 6), lies in its chemical composition. While the outer glass (the shell) can be described as sodium natron, the glass forming the inner part of the beads can be defined as the potassium ash class, due to the high content of K_2_O (up to 12%; No. 11) and the presence of phosphorus and magnesium (approx. 2.5% MgO), but with unusually high contents of Fe_2_O_3_ (up to 17%). The composition of these beads differs from the Polish beads discussed above.

A large number of particles and pores were also identified in the heterogeneous mass, as shown in Figure 6. SiO_2_ grains and particles with high iron contents were the most abundant here. Certain differences were seen in the composition of the outer glasses. In sample No. 10, PbO contents of up to 15% were detected. An analogy to high K_2_O glasses was found in the work of Conte et al. [36], who evaluated donut-shaped beads from the Chotin site. Chotin is a site in Slovakia (geographically adjacent to the Czech Republic), and the beads were classified as Halstatt D (c. 650–475 BC.). The authors [36] also inferred the use of wood ash, due to the identified contents of other elements typical of ash such as P or Ba. The text in [36] generally focuses on Iron Age black glass with a high Fe_2_O_3_ content (12% on average), and the authors assumed the introduction of this oxide with dark sand. As already mentioned, a high Fe_2_O_3_ content was also found in the discussed beads 10 and 11.

Higher K_2_O contents (of 9.8% on average) were also seen in Bronze Age glass artefacts from Poland. The latter were classified as LMHK in the work of Purowski et al. [32]. The authors discuss possible alkaline raw materials such as leached wood ash (glasses contain portions of MgO up to 1% and approx. 0.2% P_2_O_5_). With respect to the Na_2_O content (average 5.4%), the addition of a sodium component also needs to be considered. On the basis of the K_2_O/Na_2_O ratio of 1.5–2.5 for Polish glass, and the ratio value of 1–2 for Frattesina glass, two different sources of alkaline raw materials were suggested, meaning two independent production centers (both in Italy). If this ratio is applied to the samples from Bohemia, we obtain a ratio value of 4 (sample No. 10) and even 10.6 (sample No. 11), which even suggests further sources. However, due to the higher contents of P_2_O_5_ and MgO, the use of potash ash in its raw non-purified form can be assumed. This is also indicated by a higher CaO content of over 6%, compared to the values of up to 4% for LMHK glass (Table S1).

Another example of the likely use of potassium ash is that of the glasses mentioned in the work of [31] (Table 2). Although the glass from the La Négade area in France dates back to the 1st century BC–the 3rd century AD, (which does not correspond with our samples) it is evident that the use of wood ash can be considered much earlier than the commonly reported period of the transition of soda ash glasses to potassium ash glasses, typically dated to ca. the end of the 8th century AD [30].

The data are summarized in Table 2, and it is obvious that glasses with a high K_2_O content had already appeared from the 12th century BC onwards (i.e., the use of potassium ashes can be assumed). Differences are clear in P_2_O_5_ contents, which could indicate different levels of purification or the use of different plant types. The literature on later wood ash glass discusses tree species such as beech or oak, but also plants such as ferns. The chemical composition of potassium ash is highly variable, which is, of course, reflected in the resulting glass as well [48,49].

**Table 2:** Examples of Selected Oxides Represented in Potassium Glasses.

Sample	Na_2_O	MgO	Al_2_O_3_	SiO_2_	P_2_O_5_	K_2_O	CaO	Note
10	2.88	2.85	2.68	53.58	1.63	11.43	7.66	This study
11	1.17	2.42	2.26	54.39	4	12.42	6.09	This study
Figure 4	1.18	0.68	n.d	71.08	n.d	16.39	2.81/8.95	14th–9th c. BC [50]
Table 2, RC 6g	1.01	1.29	1.61	68.6	0.72	18.2	4.02	12th–10th c. BC [36]
Table 2	0.1–1	2–5.3	1.3–37	56–61	1.7–4.6	10.2–14.2	5.3–10.2	7th–5th c. BC/Chotín [36]
Table 2, LN 3	1.3	3.3	3.0	53.6	3.4	6.8	23.3	1st BC–2nd c. AD/La Négade [31]

#### 4.2.1. Non-Opaque Glasses

Artefacts made of translucent glass are also represented in the set analyzed. A large number of the beads are blue in color and made of natron glass. Certain exceptions based on K_2_O and MgO contents have already been discussed, earlier in the text. Compared to the approximately 8% CaO found in the other blue glass samples, a higher CaO content was observed in sample No. 8 (11.2% CaO). The evaluation based on the Al_2_O_3_ content is interesting, with its values being mostly above 2%, often even approaching 3%. Only sample Nos. 3, 5, and 7 have a lower content of Al_2_O_3_ (in the range of 1.24–1.8%). Sample No. 3 differs from the composition of the majority of blue glass samples with its already discussed higher K_2_O content and a rather lower level of CaO (5.7%). Similarly, the bead from Chotýš (No. 7) displays a lower CaO content. The introduction of aluminum into glass is typically associated with silica-based raw material, and thus, with regard to the overall composition, different production sites can be inferred. This is obvious, for example, in sample No. 5, where Al_2_O_3_ is present in a smaller amount (1.35%), while, at the same time, it contains up to 0.25% TiO_2_ but only 0.23% Fe_2_O_3_ (compared to the other blue glasses, where the Fe_2_O_3_ content is typically in fractions of a per cent). In fact, iron and titanium are other elements whose introduction may also be related to the silica-based raw material used.

In sample No. 4, tin-rich particles were found sporadically. Tin-based opacifiers are associated with the 4th century BC [51]. Inclusions with tin contents in beads dated to the HaD period (c. 600 to 400 BC) are mentioned, for example, by Purowski [37]. Their occurrence is associated with the opacity of white glass beads, and the author refers to another such early occurrence of SnO_2_ in glass (7th–6th cent. BC; Black Sea coast).

When assessing the blue coloration of the glass samples, a synergistic effect of the represented elements, such as blue-staining cobalt and copper coupled with the detected iron and manganese, can be assumed. In the whole set, there is an evident increase in the iron content of the blue glasses, which is probably related to the raw material used to produce this color. Elements such as copper, cobalt, and iron are commonly found, e.g., in blue glass dating back to ca. 750–400 BC in the territory of neighboring Poland [37].

**Figure 7:**
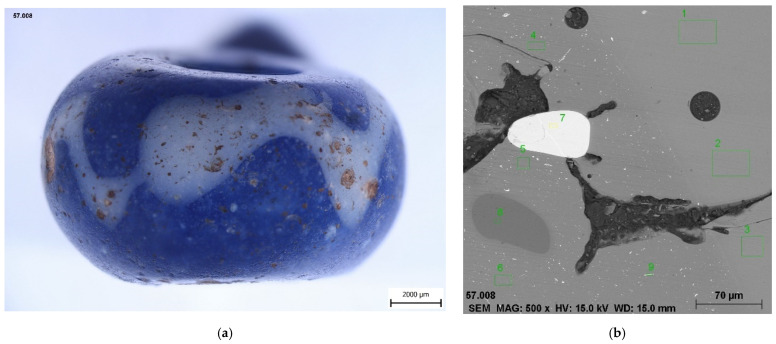
Bead No. 9 with white wavy line. (**a**) Inclusions of white color also visible in the surface layer of blue glass. The particles are antimony-based (as confirmed by Raman spectroscopy); (**b**) Interface of blue (right) and opaque white glass (left). Smaller particles of CaSb_2_O_6_, fused quartz grains (8) and particles with a likely high Zr content (7) are visible in the white glass; SEM/EDS.

Only two blue beads were further decorated with additional glass. In sample No. 9 (Figure 7), this applies to a white wavy line, while, in the case of sample 6, the ornament has mostly disappeared, probably due to corrosion processes and/or improper production technology. The glass forming the white wavy line (in sample No. 9) was made opaque with antimony-based particles (CaSb_2_O_6_; Raman band 670 cm^−1^ confirmed by Raman spectroscopy). The occurrence of particles of the white glass opacifier is lower compared to other white opaque glasses; see further in the text. Corresponding bands of CaSb_2_O_6_ phase were also found in the places of the original decoration in sample No. 6. Identical opacifiers were also found in the zigzag ornament of blue beads found at the Wicina site; dated HaD [37].

In the group of translucent/non-opaque glasses, there are also two greenish beads (specimen Nos. 30 and 29) and one colorless artefact (31). All these samples have very low Al_2_O_3_ contents (up to 0.7%) and rather high contents of SiO_2_ (over 70%) when compared to other objects from the evaluated set. In addition, Sb_2_O_3_ (0.6%) was found in sample No. 31.

An analogy to bead No. 30 can be found in the text by Purowski (ref. [52]—Figure 2, bead Wicina 95), where the glass described is very similar, not just visually, but also chemically (Table 2; 0.34% Al_2_O_3_ and 70% SiO_2_). The 3.36% Sb_2_O_5_ content of this sample, which is not opaque, is also interesting. The use of antimony compounds to decolorize several Hallstatt bead samples is suggested in the work of Purowki et al. [37].

#### 4.2.2. Opaque Glasses

A large part of the group of opaque glasses is made up of beads with eyes. The predominant type of these beads has a yellow core decorated with blue-white eyes. A group with a core in various shades of blue to green–blue is represented to a lesser extent (sample Nos. 12 and 14). From the viewpoint of chemical composition, the glasses can again be classified as natron glass, but the differences are noticeable, especially with regard to the associated opacity, as described further on in the article.

In order to describe the color of the studied beads, Raman spectroscopy measurements were performed on the samples selected. This method was used to determine the presence of opacifiers in the glass, which have a double effect: they cause the glass to turn opaque and at the same time they color it. The synergistic effect of the glass color and the opacifier used is therefore employed to obtain the final color; see e.g., [53].

##### White-Colored Glasses

**Figure 8:**
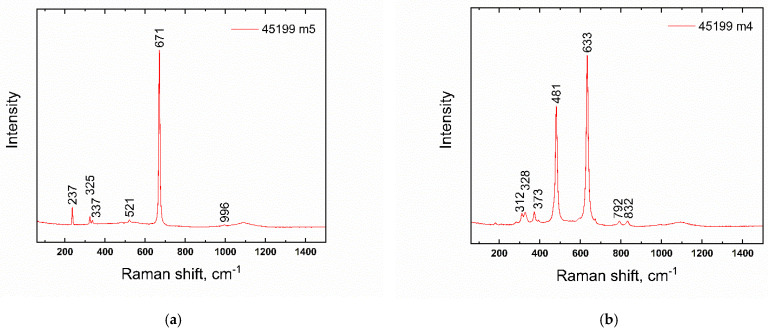
Raman-spectra of calcium antimonates; bead No. 21. (**a**) CaSb_2_O_6_; (**b**) Ca_2_Sb_2_O_7_.

In most of the white-colored glasses, the majority of opacifying agents detected were CaSb_2_O_6_-based (Raman band 670 cm^−1^; Figure 8a), often in combination with Ca_2_Sb_2_O_7_ (Figure 8b), Raman bands ~480 and 633 cm^−1^ [53,54]. White glass is represented in the set examined, both as part of the decoration, the so-called eyes applied to the surface of the yellow beads (sample Nos. 16–28), and also in the form of the wavy line seen on the blue beads (e.g., Nos. 9 or 6, already discussed earlier).

Antimony-based opacifiers were applied for a very long time to make opaque glass and were associated with the production of glass in Egypt since as early as 1570 BC, i.e., during the reign of the 18th Egyptian dynasty [55]. In the available literature, two methods of producing opaque glass are described. The first method involves the addition of natural raw material or ex situ prepared solids to the glass mass [55]. The second technique lies in the formation of crystals directly in the glass, after the addition of antimony in the form of oxide or sulfide (crystallization of calcium antimonate crystals from molten glass). Other methods, such as adding heavily opaque glass as a raw material for coloring the molten glass mass, are actually based on the two above-mentioned procedures. As a raw material for the synthesis of ex situ prepared calcium antimonate crystals, roasted stibnite in combination with calcium carbonate is usually reported. The processing temperature is estimated to have been conducted at 1000–1100 °C [53,55].

In their work, Vandini et al. [53] adopted a hypothesis that the uneven distribution of opaque crystals (in the so-called rosary-shaped conformation), and perhaps even the residues of glassy sand grains, suggest the use of an ex situ technique, while the uniform distribution of small euhedral crystals suggests an in situ crystallization technique. Several studies exist evaluating the shape of the crystalline particles formed in relation to the time and temperature of in situ crystallization, e.g., [53]. The results published there indicate that the presence of solely a hexagonal CaSb_2_O_6_ phase points to temperatures higher than 1100 °C. However, with the simultaneous presence of CaSb_2_O_6_ and orthorhombic Ca_2_Sb_2_O_7_, lower temperatures can be considered [53].

**Figure 9:**
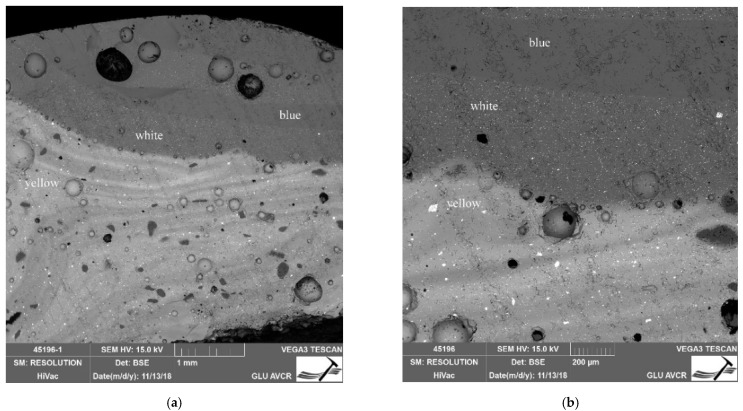
Glass Layer Stratigraphy of Bead No. 20. (**a**) Yellow glass bead decorated with white and blue eyes. The structure of yellow glass (bottom) is clearly visible, with distinct grains of SiO_2_; (**b**) detail of the previous image (**a**). The distribution of opacifiers is more uniform in the white glass and their particles are smaller than those found in yellow glass. At the color interface, a band of bubbles is clearly visible-created when the decoration was applied on the already cooled surface of the base bead.

The opacity of glasses with calcium antimonate is also discussed by Shortland [56], who, in his work, assesses the particles of the antimonate opacifier as being smaller, around 5 μm, but well distributed in glass, and containing rather exceptional clusters of such crystals. It is further argued that, in the cases where the CaO content of the opaque glass is lower than that of the translucent glass, it can be inferred that calcium was “consumed” during in situ crystallization, when only an antimony-containing raw material (in the form of oxide or sulfide) had been introduced. If crystals of complete calcium antimonate are introduced into translucent glass, an increase in the CaO content of the glass can be expected. In our set, rather lower CaO contents were detected in the white glass, compared to the non-opaque blue glass (the highest difference was, for instance, in sample No. 26, where 7.7% CaO was seen in the white glass compared to 10% CaO found in the blue glass). The distribution of the opacifiers was mostly uniform in the beads studied (Figure 9), and thus we concluded that the production technique applied for their production had been in situ crystallization.

##### Yellow-Colored Glasses

**Figure 10:**
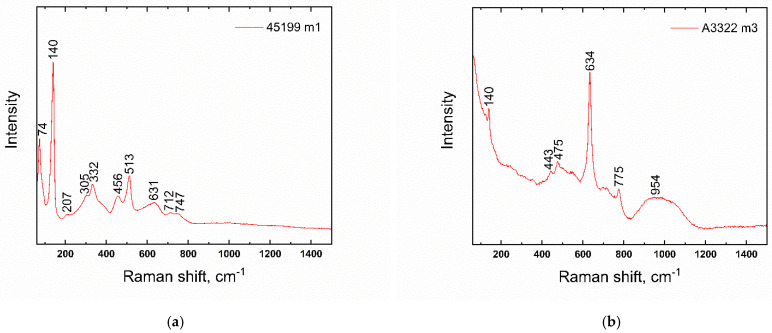
Raman-spectra of opacifying agents. (**a**) Pb_2_Sb_2_O_7_ bead No. 21; (**b**) SnO_2_; bead No. 32.

Another antimony-based opacifier is Pb_2_Sb_2_O_7_, which colors the glass yellow [53]. The Raman spectroscopy method was used to determine the yellow color in selected samples (Table S1 and Figure 10a), where the determining bands are 140, 332, and 513 cm^−1^. The occurrence of pure Pb_2_Sb_2_O_7_ in nature is rather rare, and so the reuse of antimonial litharge (derived from the cupellation of silver) and utilization of lead/antimony minerals are often cited as possible sources of the raw material. The actual opacifying technique is the topic of an ongoing debate; however, the most accepted and recognized method is an ex situ synthesis of lead antimonate and its addition to transparent glass [53,56]. For the synthesis, the combination of ores containing lead and antimony is assumed, e.g., the roasting of galena (PbS) and stibnite (Sb_2_S_3_) with an excess of lead [38,53,56].

**Figure 11:**
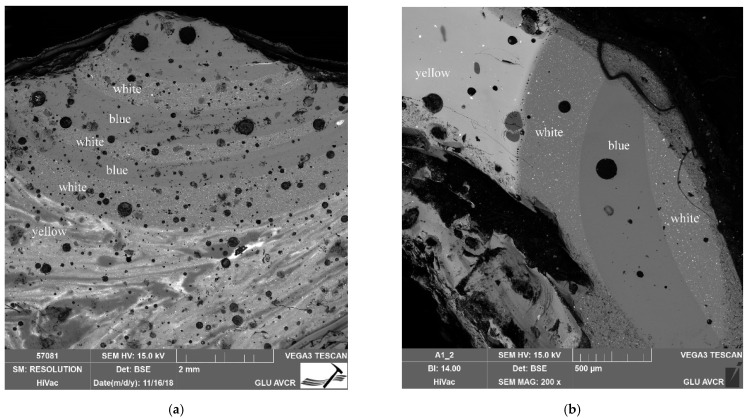
Glass Layer Stratigraphy of bead No. 23 and 26. (**a**) Layer stratigraphy, again showing highly inhomogeneous yellow glass and a large number of bubbles in all types of the glass represented; (**b**) In the case of bead No. 26, the yellow glass is relatively homogeneous (lighter area on the far left) with apparent partial dissolution of the opacifier.

Shortland [56] further states that Pb-antimonate was introduced directly into the glass melt, rather than the batch (based on the inhomogeneity of the glass and the shape of the particles). It is the appearance of streaks in the glass with the poor distribution of solids that indicates the addition of antimonate to the melted glass, followed by a subsequent insufficient mixing. Experimental data suggest that the addition of Pb-antimonate to the glass melt results in its dissolution, which can be prevented by adding the opacifier to an already cooler, and thus more viscous, melt. The distribution of particles is then irregular, explaining the occurrence of the bands with differing particle distributions, which was also observed in the case of the samples studied in this work (Figure 9 and Figure 11a). On the other hand, in the case of bead No. 26, the partial dissolution of the opacifiers used had probably already occurred, because the glass is more or less homogeneous, displaying no streaks (Figure 11b). Shortland [56] further describes rare crystals of a zonal structure, inferring their further growth in the melt during cooling. A PbO/Sb_2_O_5_ ratio higher than ca. 5 (in the glass analyzed) compared to the value of this ratio seen in the lead antimonate phase (1.38) suggests the introduction of additional lead raw materials (also Shortland [56]). Higher ratios were also found in the yellow glasses from our set. The trend of a higher PbO content (up to 17%) and a simultaneously lower Na_2_O content (13% on average) compared to the other glasses represented (white and blue; observed in our work) was also noted in the work of Purowski et al. [52], evaluating similar glass beads dated to the same period. The increased content of PbO is also interesting in the case of some samples of white opaque glass, e.g., 4% PbO in sample No. 27, 5% PbO in sample 28, and even 15% PbO in bead 15, compared to the content of mostly up to 1% PbO in the other white glasses in our set. In this context, it should be noted that, within the yellow glasses analyzed, bead 27 was found to contain one of the highest PbO contents. It is likely that glassmakers were aware of the advantage of deliberately adding lead raw material to the glass melt (to facilitate the production process of opaque glass).

##### Red-Colored Glasses

**Figure 12:**
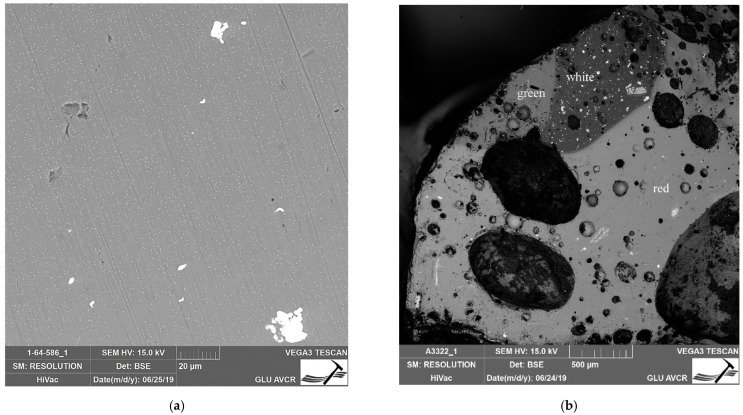
Structure of red beads. (**a**) Distribution of opacifiers in glass of Sample No. 33, two types of different sizes can be observed; (**b**) Heterogeneous structure of Sample No. 32, a red bead with a white (darker area at the top of the image) and green line on the surface.

Within our set, only two beads (32 and 33) had a unique red color. The glass of both artefacts is also opaque and opacifying agents were identified as being based primarily on copper and also tin. As regards the single-colored bead 33, an SEM image (Figure 12) revealed a noticeable representation of larger particles up to about 20 μm (rich in Sn, we assume the form of SnO_2_), as well as smaller crystals in the order of micrometres. In this particular case, it is Cu° particles (as confirmed by microXRD) coloring the glass red.

The structure of the second red bead decorated with white and green glass (No. 32) is highly heterogeneous, see Figure 12b. The image shows large bubbles (even in hundreds of μm), especially in the red glass and the shape and the quantity of particles in individual glasses. The presence of an SnO_2_-based opacifier was confirmed by Raman spectroscopy in all the represented colors (red, white, green; Figure 10b). Clearly, SnO_2_ was involved in coloring the white glass mass. In the case of the green glass, an obvious increase can be seen in CuO and Fe_2_O_3_ contents. As for the red glass, in addition to the discussed SnO_2_, iron-rich particles (iron oxides confirmed by micro-XRD) and titanium were found. The work in [57], for instance, attributes the combination of these two elements (Fe and Ti) to their introduction when using an iron-rich raw material in the context of glass coloring.

An analogy to this sample can be found in the work of Boscheti et al. [57], where an apparently identical sample is presented with the designation LEMA 61. A tin-based opacifier was detected, both in white and green glass. Bead No. 32 is similar to the sample in the discussed work, in that it shows a higher PbO content (more than 7%; applying to green and red glass), while the white glass has a lower content. The work in [57] evaluates red glass as a Foy 2.1. type, and the other groups as Roman glass (this evaluation was based on the comparison of TiO_2_/Al_2_O_3_ and Al_2_O_3_/SiO_2_ ratios used in the characterization of Roman and younger glasses). The Foy 2.1 type is believed to be of Egyptian origin, and was widespread in Europe and North Africa from the second half of the 5th to the 7th centuries AD [58]. It seems that bead No. 32 is of a younger date than the other beads in the set. Furthermore, iron-based particles were detected in the red glass. Similarly, particles of the Fe_3_O_4_ and Fe_2_SiO_4_ types were found, for example, in a brick-colored bead from the Migration Period (authors’ archive), which again suggests its younger origin.

##### Dark-Colored Glass

The last and rather unique opaque bead is a dark-colored bead with white and yellow eyes (No. 15). Its dark glass can be characterized as sodium natron with a slightly elevated level of K_2_O (1.6%). However, elements such as Fe, Co, Ni, Cu, Sb, Pb, and As were also present in considerable amounts. SiO_2_ particles, probably even feldspar and particles with a high content of copper and other metals (Sb, Fe, Co, Ni, Cu) were detected as being scattered in the glass mass. Phases based on silicates, mixed oxides of heavy metals (bands ca. 200 and 510 cm^−1^), and copper were confirmed by Raman spectroscopy. Purowski [37,38] described similar metal inclusions in the glass mass of Hallstatt beads and considered the use of metallurgical slag in their production. The glasses were classified as LMMK with 1.4–2.2% K_2_O, which corresponds to our sample too. Cobalt in combination with nickel and arsenic is mentioned by Conte [36] as a possible indication of the use of the mineral skutterudite [(Co,Fe,Ni)As_3_] for the coloring of the glass. Particles with high Pb and Sb contents were found in the yellow glass, probably being of the Pb_2_Sb_2_O_7_ type.

## 5. Conclusions

A total of 34 beads were examined during the project. The detailed study of their chemical composition and in some cases technological specifics allowed precisely dating the selected beads. The composition of the analyzed sample set, being diverse from the typological and technological point of view, could indicate various workshop districts with different production traditions. At the same time, the identification of varying chemical compositions in the examined beads may suggest the use of raw glass from several primary workshops, the exact locations of which remain unknown. Only one sample represented the LMHK type (No. 1), which can be dated to the Late Bronze Age and could be connected with the Fratessina workshop. The most frequent type from the analyzed collection are beads with blue and white eyes made of yellow opaque glass (13 beads). The studied set was found to be dominated by sodium natron glass of the LMG type, which is typical for the Hallstatt period. Such yellow beads are well known, both from funeral and settlement contexts, and are distributed practically all over Bohemia, and in most cases are dated to the Late Hallstatt period (from the 6th until the 4th century BC). Simple rounded beads made of cobalt blue glass (Nos. 2–9.) make up the second largest group. In the case of beads Nos. 5–6, 8–9, it is possible to consider a later dating, to the Late Iron Age (see Supplementary Materials); however, these beads also belong to the LMG type. A simple bead from Bylany (No. 3) stands out from these groups, with a higher K_2_O (1.84%) content and a rather lower level of CaO (5.7%). A rather unique type in the set is represented by bead Nos. 10 and 11, which were determined to be made of glass with a relatively high K_2_O content. Their representation in the sample set is quite low (2 beads), but the occurrence of this glass has been described in Europe, indicating the use of potassium raw materials already in such an early period. It needs to be emphasized that this type of glass does not correspond to the mixed-alkali ash glass from the Italian region. Given the inferior quality of the glass (unmelted particles and bubbles present), but also the potassium content, it is necessary to consider its production outside such areas as the eastern Mediterranean, where glass production was carried out to a superior level. Beads 10 and 11 also contain an interesting “double layer” structure. This technique was well known in the later periods (namely, the La Tène and the Migration Period), but in relation to the Hallstatt period in Central Europe it represents yet another interesting objective for future research.

Bead No. 15 comes from the eponymous site of Platěnice and stands out from the group with a specific decoration of three eyes with rows of three dots. Its dark glass can be characterized as LMMK with a slightly elevated level of K_2_O (1.6%). Beads with similar decorations belong to rather rare finds in the Bohemian region; however, comparable beads are known from sites in Moravia and Poland. Glass ring No. 31 belongs to the specific find from Závist hillfort. The ring was originally dated to the Late Hallstatt period. The presence of Sb_2_O_3_ (0.6%) attests to the use of antimony compounds to decolorize glass during the Hallstatt period. The chemical analyses confirmed that several glass beads could be dated to later periods. Two red colored beads (Nos. 32–33) are assumed to be of a younger date; from the Migration period. The amber brown bead No. 34 stands out not just for its color, but also for its production technique using a mold. A specific chemical composition (higher K_2_O 14.7% and lower Na_2_O 3.61%) also indicates the Medieval or Modern period.

## Figures and Tables

**Table 1:** List of the analyzed glass beads.

Number	Inventory/Museum Number	Site	Basic Description	Find Context	Chronology	Type
1	H1–111324	Křepice hillfort	fusiform/cylindrical bead with spiral decoration	settlement, accidental find	Ha B (1000–800 BC)	pfahlbauperle/type 4 after Venclová 1990
2	H1–46623	Prague, Střešovice	blue rounded bead	necropolis, inhumation grave	Ha C (800–600 BC)	type 119 after Venclová 1990
3	H1–110925	Bylany	blue rounded bead	necropolis, grave №16/1897	Ha C (800–600 BC)	type 121 after Venclová 1990
4	H1–45060	Prague, Bubeneč	blue annular	necropolis, cremation grave №1/31	Ha D (600–450 BC)	type 155 after Venclová 1990
5	H1–111320	Křepice hillfort	blue rounded	settlement, accidental find	Ha C–LT (800–50 BC)?	type 119 after Venclová 1990
6	H1–111321	Křepice hillfort	blue with wavy line	settlement, accidental find	Ha C–LT (800–50 BC)	type 708–710 after Venclová 1990
7	UAPPSČ	Chotýš	blue annular	settlement	Ha D2–LT A (550–380 BC)	type 155 after Venclová 1990
8	A 4471 a	Lhota–Závist hillfort	blue rounded	settlement, acropolis	Ha D2–LT A (550–380 BC)	type 119 after Venclová 1990
9	H1–57008	Mydlovary	blue with white wavy line	?	Ha C–LT (800–50 BC)?	type 707 after Venclová 1990
10	A 4471 b	Lhota–Závist hillfort	blue rounded, double layer	settlement, acropolis	Ha D2–LT A (550–380 BC)	type 119 after Venclová 1990
11	H1–15394	Černuc	blue rounded, double layer	cremation grave ?	Ha D (600–450 BC)?	type 119 after Venclová 1990
12	C 214	Lhota–Závist hillfort	blue-green with compound eyes	settlement, acropolis	Ha D2–LT A (550–380 BC)	type 549 after Venclová 1990
13	F 390	Lhota–Závist hillfort, gate D	translucent blue flattened globular bead with spiral decoration	settlement	Ha D2–LT D (550–380 BC)	type 414 after Venclová 1990
14	H1–26276	Žalov	blue with blue-white eyes	?	Ha C–D (800–450 BC)	type 520 after Venclová 1990
15	H1–225671	Platěnice	blue with yellow circules	necropolis, cremation grave №60	Ha C (800–600 BC)	type 552 after Venclová 1990
16	H1–26286	Žalov (u Prahy)	yellow with blue-white eyes	?	Ha C–D (800–450 BC)	type 533 after Venclová 1990
17	H1–26773	Prague, Nové Město	yellow with blue-white eyes, deformed	?	Ha C–D (800–450 BC)	type 533 after Venclová 1990
18	H1–26922	Pátek	yellow with blue-white eyes	inhumation graves?	Ha C–D (800–450 BC)	type 519 after Venclová 1990
19	H1–26923	Pátek	yellow with blue-white eyes	inhumation graves?	Ha C–D (800–450 BC)	type 519 after Venclová 1990
20	H1–45196	Prague, Bubeneč	yellow with blue-white eyes	necropolis, inhumation grave	Ha D (600–450 BC)	type 533 after Venclová 1990
21	H1–45199	Prague, Bubeneč	yellow with blue-white eyes	necropolis, inhumation grave	Ha D (600–450 BC)	type 533 after Venclová 1990
22	H1–45200	Prague, Bubeneč	yellow with blue-white eyes	necropolis, inhumation grave	Ha D (600–450 BC)	type 539 after Venclová 1990
23	H1–57001	Lochovice	yellow with blue-white eyes and yellow prunts	burial mound	Ha D2–LT A (550–380 BC)	type 548 after Venclová 1990
24	AS 18/3/3	Holubice	yellow with blue-white eyes and yellow prunts	settlement	Ha D2–LT A (550–380 BC)	type 548 after Venclová 1990
25	H1–57005	Rtišovice	yellow with blue-white eyes	?	Ha C–D (800–450 BC)	type 519 after Venclová 1990
26	H1–40054	Láz	yellow with blue-white eyes	necropolis, mound IX/1922	Ha D2/Ha D3 (550–450 BC)	type 531 after Venclová 1990
27	A 3031	Lhota–Závist hillfort	yellow with blue-white eyes	settlement, acropolis	Ha D2–LT A (550–380 BC)	type 519 after Venclová 1990
28	A 4309	Lhota–Závist hillfort	yellow with blue-white eyes, deformed	settlement, acropolis	LT A (450–380 BC)	?
29	H1–111202	Straškov	green rounded	inhumation grave	Ha C (800–600 BC)	type 132 after Venclová 1990
30	H1–600070	Minice hillfort	green rounded	settlement	Ha D (600–450 BC)	type 132 after Venclová 1990
31	F 1762	Lhota–Závist hillfort	ring colourless	settlement, gate D	Ha D (600–450 BC)	type 34 after Venclová 1990
32	A 3322	Lhota–Závist hillfort	larger rounded bead with line decoration	settlement, acropolis	Migration period (380–570 AD)	–
33	1/64_586	Lhota–Závist hillfort	cylindrical bead	settlement, acropolis, secondary position	Migration period (380–570 AD)?	type 147 after Venclová 1990
34	H1–111322	Křepice hillfort	orange rounded	settlement, accidental find	recent	type 124 after Venclová 1990_amber

## Data Availability

Not applicable.

## Supplementary Materials

The following supporting information can be downloaded at: https://www.mdpi.com/article/10.3390/ma15165740/s1, Table S1: Chemical Composition of Glasses Examined; in wt.%. The number of analyses is higher than the number of samples because all the types of the glass found in one bead were measured, (abbreviation: n.d. = not detected).
