# Supplementary material for: Chemistry and Production Technology of Hallstatt Period Glass Beads from Bohemia
Source: Materials (Basel). 2022 Aug 19;15(16):5740. doi: 10.3390/ma15165740 (PMC9414364; doi:10.3390/ma15165740)
Supplement: Supplementary file 1 [file materials-15-05740-s001.zip › materials-1756085-supplementary.pdf]

materials

(Supplementary Materials)

Fusiform/cylindrical bead No. 1 is made of a dark blue (almost black) glass with traces of a spiral decoration. This bead belongs to the so called Pfahlbauperlen group dated to the Late Bronze Age (11th - 10th centuries BC). Bead No. 1. belongs to the group of glass finds from a prehistoric hillfort in Křepice. From Moravia, other beads of the same type are known, for example, the beads from the sites Brno-Obřany and Blučina. Three specimens of the Pfahlbauperlen group are also documented from the sites of the Knovíz culture found in Bohemia, namely in Řepín and Tuchoměřice ([4,5]). The Pfahlbauperlen group is also described in the work of Haevernick [6], where the author allocated the main occurrence of this type of beads to present-day Switzerland and north Germany. The production of such beads could be connected with a north Italian site in Frattesina, where the presence of a glass workshop was recorded. New data from this site seem to support the idea of local glassmaking production [5]. Based on the typology and specific chemical composition (see below), bead No. 1 can be dated to the Late Bronze Age.

One of the most common bead types in the context of Bohemian prehistory is that of rounded beads made of translucent cobalt blue glass (numbers 2–11.). Rounded and undecorated beads of various shades of blue and different sizes make up a large group that started to appear in the Bronze Age and disappeared during the Migration period. Smaller dark cobalt blue beads (with a diameter around 10 mm) are characteristic of both the Late Hallstatt and La Tène periods [4,7]. Samples No. 6 and 9 are dark blue beads decorated with a wavy line. These beads belong to a large subgroup of rounded/annular blue beads with wavy decorations. Wavy decorations started to be popular during the Late Bronze Age, reaching a peak in the Hallstatt period (Ha C - Ha D) and appearing from north Germany to Italy and also in the territory of the former Yugoslavia [8]. Beads with white or yellow wavy lines are also known from the La Tène period; however, they differ in that they have a lighter cobalt blue glass [7].

Cobalt blue bead No. 13 (type 414) belongs to a subgroup of beads with a single-colored spiral decoration. This bead belongs to a less frequent type of the finds from the Czech Republic. The find context is unclear. The bead was uncovered in the Late Hallstatt hill fort called Lhota–Závist, in the context of gate D. The finds of beads with spiral decorations are spread from the Mediterranean to central Germany, with the earliest examples dating to the Late Bronze age. These beads primarily date to the Late Hallstatt period; the 6th century BC. On the other hand, it is worth mentioning that a group of beads with a similar decoration exists but was dated to the Middle La Tène period. One bead close to our example is known from a Moravian Hallstatt Lusatian culture necropolis in Moravičany. Another parallel is a green bead with three white spirals from Býčí skála, dated to the period Ha D2/Ha D3 [4]. Therefore, bead No. 13 from Lhota–Závist could be dated to the Late Hallstatt period, 6th–5th century BC.

Bead No. 15 comes from the eponymous site of Platěnice, where an extensive burial site of the Silesian-Platěnice culture was found. A necklace from grave 60 contains 12 similarly sized beads made of a dark blue opaque glass. The beads have almost identical decorations of three ringlets with rows of three dots placed between them [9,10]. Typologically, the bead can be assigned to group 552 according to Venclová [4]. Beads with similar decorations have rarely been found in the Bohemian region. However, comparable beads are known from sites in Moravia: the Moravičany burial site [11], Brno, Modřice - Sádky [12], Slatinky-Nivky [4], and Příbor [13]. Beads close to this type are also recorded at the German site of Magdalenenberg [14] or in Sopron, Hungary [8]. Moreover, an abundant occurrence of dark blue beads decorated with yellow/white ringlets is documented from Poland [4]. On the basis of a preliminary analysis of the grave goods from grave No. 60 in Platěnice, bead No. 15 can be dated to the Ha C phase. Several finds from Moravia (e.g., Brno, Modřice-Sádky) can also be dated to the later Ha D1 phase, which allows us to date

the occurrence of these beads in Central Europe to the period from the 8th to the turn of the 7th and 6th centuries BC [9].

Beads with blue and white eyes belong to the most frequent glass bead type in the Early Iron Age. The complex classification of such beads was devised by N. Venclová [4], who distinguished 26 types. The classification of the beads depends on the number of eyes found on the particular bead and the number of eye layers. The shapes typically range from irregularly cylindrical to barrel-shaped. The predominant material used is opaque glass, whereas translucent glass occurs only in combination with blue glass [4]. In our collection, we can distinguish beads made of translucent blue glass decorated with blue and white eyes (No. 14), but the largest group is represented by beads made of opaque yellow glass, again with blue and white eyes (Nos. 16 - 28). Such yellow beads are known both from funeral and settlement contexts, and are distributed practically all over Bohemia. Here, we can mention, in particular, the Late Hallstatt period hill fort Lhota–Závist [15], an early La Tène settlement in Dolní Břežany, and Late Hallstatt hill fort Svržno [16]. The majority of the eye beads in Bohemia can be dated to the Late Hallstatt period Ha D (after the end of Ha D1) to LT A; from the 6th until the 4th century BC. Beads found later than LT A are quite rare. The secondary use of these beads cannot be excluded for later occurrences [4].

Bead No. 12 from the hill fort Lhota–Závist belongs to a rather specific type. The bead is made of a blue-green glass and is decorated with compound eyes; type 549 "Zusammengesetzte Auge". These beads are widely spread across Europe, from England to China, with the main concentration being in the north Black Sea region. Another distribution area is related to the territory of Italy, Greece, and the Balkans. The main occurrence of these beads is dated to the 6th to the 4th centuries BC [4,17].

A specific subgroup is created by two fragments (No. 23 - 24) of larger beads with blue-white eyes and small prunts. The most well-known finds of this type in the region of the Czech Republic are seven large eye beads from Vicenice. The type in question is well known from the Mediterranean and the northern area of the Black Sea, but only a few examples have been recorded in Central Europe [17]. Beads of this type are dated to ca. the Ha D/LT A; from the 6th century to the end of the 5th century BC; [4,18].

Numbers 29 and 30, which are larger rounded beads, constitute another subgroup. This type of bead is well known from the territory of Central Europe, both from funeral and settlement contexts. Larger rounded beads of different colors are abundantly present in the collection from the site of Býčí skála [19]. Similar beads have also been recorded at various sites dating from Ha C to Ha D/LT A. However, large rounded beads were popular during the La Tène and Roman periods as well, particularly until the 1st century AD [4].

Fragment 31 can be identified as a part of a glass ring. The find can be assigned to a rather small group of glass rings from the Late Hallstatt period. The find from the hill fort Lhota–Závist is characteristic, with a translucent colorless glass with a light yellowish tint and an obvious trace of the folding of a glass rod, which is a typical element in the aforementioned rings from the Late Hallstatt period [7]. Similarly shaped rings are reported from a region in north-eastern Italy, from where the tradition of such production is thought to have originated. Other finds are also documented in Switzerland, Baden-Württemberg, Bavaria, Thuringia, and the central Rhineland, while other specimens come from southern and eastern France [4,20]. Glass rings are also known from Bohemia, where they are mostly dated to the Late Hallstatt Age Ha D2/D3. A detailed analysis of the Czech finds is presented in Chytráček et al [21]. It should also be mentioned that glass rings are not only a feature of the Late Hallstatt period. Finds of a similar shape can be seen in collections coming from the La Tène period as well [7], while colored glass rings were also very popular in the Middle Ages, with their popularity peaking in the period from the 11th until the 13th centuries ([10] Abb. 6, [22]).

Bead No. 32 stands out in the sample set due to its unusual coloration, which was not common in the Late Hallstatt period. The bead has a terracotta red core with green and

white wavy lines. A similar bead comes from the burial site of Aschheim-Bajuwarenring dated to the Migration Period. There, a similar type of bead was found in graves 368 and 238 dating to the 6th century AD. The sites of Keszthely-Fenékpuszta or Pleidelsheim are also worth noting, as they contained beads of similar typology, coming from grave sets dated from the end of the 6th century to the 1st third of the 7th century AD [23,24].

Cylindrical bead No. 33 is made of opaque dark red glass, which was particularly popular during the Migration period [25]. Typologically close parallels are documented in the sets of finds, for example, from the sites of Keszthely-Fenékpuszta and Aschheim-Bajuvarenring [26,24]. A similar cylindrical bead of an opaque blue glass is recorded in the burial site of Holubice dating back to the 6th century AD [27]. Opaque red cylindrical beads were also uncovered in a rich female grave from Smolin, which is, however, dated to the middle of the 5th century AD [28]. Here, it is necessary to mention that cylindrical beads made of red opaque glass are also known in relation to later periods: the Middle Ages and the Modern era. Beads No. 32 and 33 were originally identified as Late Hallstatt and were somewhat surprisingly assigned to the North Sea production [15]. It is notable that traces of a settlement dating from the end of the 4th to the 6th century AD have also been detected at the Lhota–Závist site [29]. Owing to the specific composition (see below) and typological determination, the beads must be placed in the later period, the end of the 4th–6th century AD; the period of the migration of nations.

Bead No. 34 comes from the already mentioned prehistoric settlement of Křepice. It should be mentioned that just on the basis of the typological and visual assessment of the glass mass, this bead appeared to be a recent find, which was originally misidentified as a Hallstatt glass bead. This assumption was subsequently confirmed by the elemental composition survey (see below).
